# Comparison study of reconstruction algorithms for volumetric necrosis maps from 2D multi-slice GRE thermometry images

**DOI:** 10.1038/s41598-022-15712-7

**Published:** 2022-07-07

**Authors:** Julian Alpers, Bennet Hensen, Maximilian Rötzer, Daniel L. Reimert, Thomas Gerlach, Ralf Vick, Marcel Gutberlet, Frank Wacker, Christian Hansen

**Affiliations:** 1 Faculty of Computer Science, Otto-von-Guericke University, 39106 Magdeburg, Germany; 2 Faculty of Electrical Engineering and Information Technologies, Otto-von-Guericke University, 39106 Magdeburg, Germany; 3 Institute for Diagnostic and Interventional Radiology, Hannover Medical School, 30625 Hannover, Germany; 4 Research Campus STIMULATE, Otto-von-Guericke University, 39106 Magdeburg, Germany

**Keywords:** Software, Computational science, Computer science, Cancer therapy

## Abstract

Cancer is a disease which requires a significant amount of careful medical attention. For minimally-invasive thermal ablation procedures, the monitoring of heat distribution is one of the biggest challenges. In this work, three approaches for volumetric heat map reconstruction (Delauney triangulation, minimum volume enclosing ellipsoids (MVEE) and splines) are presented based on uniformly distributed 2D MRI phase images rotated around the applicator’s main axis. We compare them with our previous temperature interpolation method with respect to accuracy, robustness and adaptability. All approaches are evaluated during MWA treatment on the same data sets consisting of 13 ex vivo bio protein phantoms, including six phantoms with simulated heat sink effects. Regarding accuracy, the DSC similarity results show a strong trend towards the MVEE ($0.80\pm 0.03$) and the splines ($0.77\pm 0.04$) method compared to the Delauney triangulation ($0.75\pm 0.02$) or the temperature interpolation ($0.73\pm 0.07$). Robustness is increased for all three approaches and the adaptability shows a significant trend towards the initial interpolation method and the splines. To overcome local inhomogeneities in the acquired data, the use of adaptive simulations should be considered in the future. In addition, the transfer to in vivo animal experiments should be considered to test for clinical applicability.

## Introduction

From 2018 to 2020, the incidences and deaths caused by cancer increased by 6.47% and 4.21%, respectively^1,2^. Minimally invasive approaches are considered safer than open surgery. In addition, open surgery is not always applicable due to comorbidities, the spread of tumorous lesions or anatomical limitations. A growing alternative is the use of minimally invasive approaches. Along with others, microwave ablation (MWA) is a promising technique for treating primary and secondary liver lesions with several FDA-certified systems on the market^3^. MWA offers several advantages for the treatment of tumorous lesions, such as fast temperature development within the tissue, fast ablation times, and the ability to use several applicators simultaneously. In addition, MWA tends to reduce patient trauma and to increase the 5-year survival rate of patients with, e.g., hepatocellular carcinoma and liver metastases^4^. No matter what ablation technique is used, it is of greatest importance to assess if the whole lesion has been destroyed, including a minimal ablative margin (MAM). This MAM was found to be the only important predictor of local tumor progression (LTP) for liver lesions. Laimer et al.^5^ showed that the chance for LTP was decreased by 30% for each millimeter increase of the MAM. To track the temperature inside the tissue and assess the live coagulation necrosis, magnetic resonance imaging (MRI) thermometry can be performed. Using MRI, the most common approach for thermometry monitoring is the proton resonance frequency shift (PRFS) method using phase mapping^6,7^. In our previous work^8^, we introduced a novel approach for the creation of a volumetric thermometry map by utilizing common gradient-echo sequences and performing a simple spatial interpolation. Nonetheless, the approach has disadvantages. The temperature interpolation is prone to errors and anatomical inhomogeneities. Therefore, a priori knowledge about risk structures is necessary. Additionally, the evaluation of bio-protein phantoms raises another bias due to the variable threshold for the coagulation necrosis, based on the pH-value. In this work, we compare three approaches for reconstruction of a volumetric necrosis map. All algorithms are evaluated on the same data sets introduced in our previous work^8^, including updated thresholds to minimize the bias caused by the phantoms. We will show that our new approaches are able to exceed the similarity of the previous reconstruction method while being more robust towards artifacts and outliers. The goal of the evaluation is the identification of a suitable reconstruction approach with respect to the accuracy, robustness, and the adaptability (necessity of a priori knowledge).

## Related work

Previous work in the field of volumetric necrosis estimation is mainly limited to the development of unique MR sequences using either a stack of 2D images or a full 3D sequence. Apart from the spatial and temporal resolution, those approaches differ highly regarding the dimension of the acquired images (2D or 3D), the sampling of the k-space (Cartesian, Echo-Planar-Imaging (EPI) and others), and the used number of echos (Single or Multi).

**Table 1:** Overview of the related work on different approaches to create a volumetric heat map. The table is sorted with respect to the year of publication starting from 2009 to 2021 in ascending order.

	Method	Dim.	Sampling	Echo	Acq. Time [s]	Resolution [mm]	Coverage [mm]	Temp. [$^\circ$ C]	Reco. Time [s]
^9^	HIFU	2D	EPI	Single	2.9	$2.5\times 2.5\times 7.0$	$400\times 248\times 21$	$1.2\pm 0.2$	–
^10^	FUS	3D	EPI	Single	1.2	$1.5\times 1.5\times 3.0$	$192\times 162\times 66$	$\pm 0.5$	0.72
^11^	FUS	3D	EPI	Single	2.4	$2.3\times 2.3\times 2.5$	$288\times 221\times 135$	$<1.1$	–
^12^	FUS	2D	Spiral	Multi	$<=5$	$2.0\times 2.0\times 2.0$	$360\times 360\times 360$	$<0.5$	16
^13^	FUS	3D	Pseudo-Golden-Angle Stack-of-Stars	Multi	$<3$	$1.3\times 1.3\times 1.3$	$208\times 208\times 41.6$	0.3–1.0	–
^14^	FUS	3D	Stack-of-Spirals (RIO)	Single	2.9–3.3	$0.4\times 0.4\times 0.4$	$224\times 224\times 224$	1.3	2.9
^15^	HIFU	3D	Golden-Angle-Ordered Stack-of-Radial	Multi	2–5	$1.17\times 1.17\times 5.0$	$300\times 300\times 160$	$<2$	–
^16^	HIFU	3D	Cartesian	Single	3.3	$2.0\times 2.0\times 5.0$	$192\times 192\times 80$	$\pm 0.56$	–
^17^	FUS	2D	Cartesian	Single	11.7	$??\times ??\times 3.0$	–	$<1$	9.1
^18^	HIFU	3D	Cartesian	Single	3	$2.0\times 2.0\times 5.0$	$192\times 192\times 80$	0.37–0.45	–
^19^	HIFU	2D	Cartesian	Single	10	$1.7\times 2.0\times 5.0$	$150\times 150\times 25$	–	10
^20^	Laser	2D	EPI	Single	2.9	$1.44\times 1.44\times 3.0$	$300\times 300\times 24$	0.38	–
^8^	MWA	2D	Cartesian	Single	1.1	$1.0\times 1.0\times 5.0$	$256\times 256\times 256$	1	0.18

For 3D, single echo sequences can be differentiated into the use of EPI sequences^10,11^ and stack-of-spirals^14^. Multi echo sequences either use a pseudo-golden-angle stack-of-stars^13^ or true-golden-angle-ordered stack-of-radial^15^. With respect to a stack of 2D, single echo sequences can be divided into EPI sequences^9,20^ as well as Cartesian sequences^8,17,19^. Regarding multi echo, Marx et al.^12^ us a spiral sequence. A detailed analysis of the related work can be observed in Table 1. All related works have been analyzed regarding the used intervention technique, the dimension of image acquisition, the used sampling, the number of echoes used, the acquisition time of the volume, the spatial resolution of the images, the volume coverage, the temperature accuracy of the used thermometry and the reconstruction time of the image volume if available.

Our previous work^8^ utilizes a common 2D single echo Cartesian gradient-recalled echo (GRE) sequence. We showed how we are able to cover a volume of $256\times 256\times 256$ mm with the standard temperature accuracy of 1 °C. Even though the work of Marx et al.^12^ exceeds these parameters by offering a volume coverage of 360 × 360 × 360 mm and a temperature accuracy < 0.5 °C, we offer a reconstruction time of the full volume of 10.4s contrary to the 16s offered by their work. Here a single 2D image can be acquired within  1.02s using GRAPPA acceleration. The reconstruction of the whole volume is done in 0.18s for each image resulting in a full sampling of the volume in 10.4s. The acquisition time of the volumetric heat map and the possibility to use standard sequences allow for an easier and more pleasant applicability and real-time monitoring during intervention. Therefore, this work focuses on real-time capability of the reconstruction and the independence from custom MR sequences while preserving a high volume coverage and the temperature accuracy provided by 2D GRE sequences^21^.

The reconstruction methods analyzed in this work include the Delaunay triangulation, the Minimum Volume Enclosing Ellipsoid (MVEE) and Cubic Bezier Curves. Bowyer^22^ and Watson^23^ introduced an incremental 3D Delaunay triangulation. Their initial approach has a time complexity of $O(N^{3/4})$ to $O(N^2)$ and belongs to the serial strategies. Because the efficiency for large data sets is bad other approaches have emerged using parallel strategies. Marot et al.^24^ offer a 3D triangulation approach using parallel computation, which is able to reconstruct 55 million tetrahedas in one second. Su et al.^25^ provide a detailed related work analysis and another approach for a rapid 3D Delaunay algorithm adapting 3D Hilbert curve and 3D multi-grid division to extend the basic triangulation. Overall, the Delaunay triangulation is said to be suitable for reconstruction of homogeneous structures and convex surfaces.

Regarding the MVEE Van et al.^26^ offer a detailed analysis of the algorithm. They are able to show that the algorithm behaves properly under affine transformation of the data points and provides an efficient convergence rate. In addition, it is shown that the highest break down value lies at around 50%, which is said to be the maximum value for all affine equivariant estimators like the MVEE. Abo et al.^27^ on the other hand introduce the use of MVEE for a finite set of points and show that the problem can also be solved by computing the MVEE of a polytope defined by the convex hull.

The Bezier splines are an efficient approach to evaluate a spline curve at a given point while being numerically stable. De Boor^28^ also increased the efficiency by introducing the condition that no terms are computed, which are guaranteed to be multiplied by zero. Denk et al.^29^ introduced the splines to perform a myocardial displacement and strain reconstruction using a new cylindrical coordinate B-Spline model, which takes roughly 20s to compute. Galassi et al.^30^ on the other hand reconstruct the 3D coronary artery from 2D X-ray images. They use a non-uniform rational basis splines called NURBS and perform an joint operation on the 2D reconstruction to compute the 3D volume at the end.

## Material and methods

In Alpers et al.^8^, we describe a new approach for creating a volumetric heat map. We utilize a 2D GRE sequence (TE = 3.69 ms, TR = 7.5 ms, flip angle = 7$^\circ$, FOV = $256\times 256$ mm, matrix = $256\times 256$, bandwidth = 40 Hz/Px, slice thickness = 5 mm) by rotating it around the applicator’s main axis and reconstructing the missing information. Because we focus on 3D reconstruction we chose the GRE sequence inspired by Gorny et al.^21^ who also provide an in depth study about the temporal resolution of GRE sequences during the use of MWA. Therefore, our 13 bio protein phantom data sets offer a temperature accuracy of $\pm 1\,^\circ$C and each slice was acquired with a resolution of $1.0\times 1.0\times 5.0$ mm in 1.1s. In the following subsections, we briefly describe the used temperature interpolation method as well as the three new developed approaches for volume reconstruction (Delaunay triangulation, MVEE , and splines). All coagulation necrosies were computed using the critical temperature model and a phantom specific threshold between $50\,^\circ$C and $60\,^\circ$C. Examples of the phantoms can be seen in Supplementary Fig. 1. For rapid prototyping purposes, all three new approaches are implemented in python. The source code can be accessed via https://github.com/jalpers/ScientificReports2022_ComparisonStudy.

### Temperature interpolation

**Figure 1:**
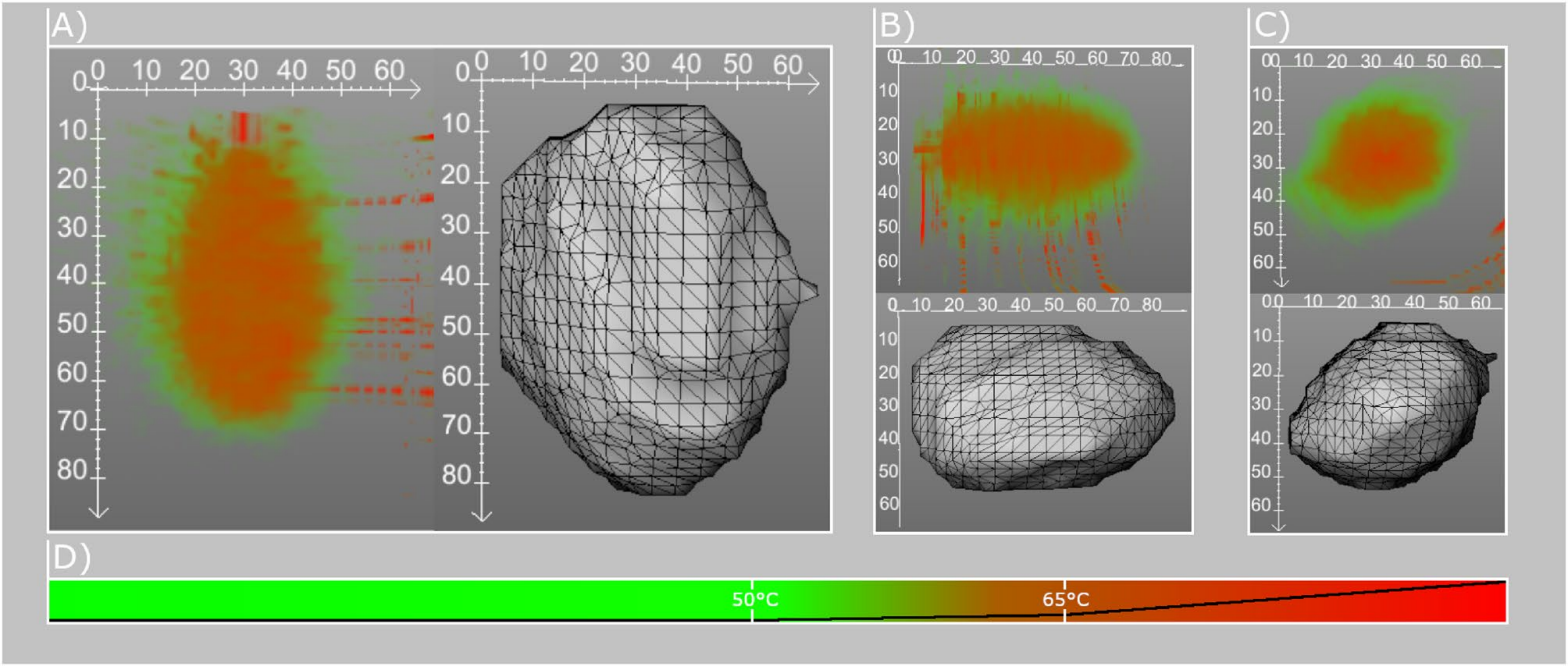
Reconstruction results for the temperature interpolation and the Delaunay triangulation in axial (**A**), sagittal (**B**) and coronal (**C**). (**D**) shows the color bar used for the temperature interpolation visualization (0$^\circ$C–100$^\circ$C). The black line indicates the alpha value of the LUT.

The temperature interpolation method aims at reconstructing the heat map and not the estimated necrosis map. Here, the Cartesian 2D coordinates were mapped to the corresponding polar coordinates. After acquisition of the phase reference images used for the PRFS method to compute the heat map, a population map is created. This population map holds the weights for each interpolation partner for every voxel. The weights are computed using Eq. (1):

1 $$\begin{aligned} w_{1}&= \left| \frac{\theta _{IP_{left}} - \theta _{i}}{\theta _{IP_{left}} - \theta _{IP_{right}}}\right| \nonumber \\ w_2&= 1 - w_1 \end{aligned}$$

with $\theta _{i}$ representing the cylindric angle of the current voxel *i* and $\theta _{IP_{left}}$, $\theta _{IP_{right}}$ representing the orientation angles of the left and right interpolation partners, respectively. The final interpolation of the temperature is done by applying the population map to every slice along the applicator’s main axis using Eq. (2):

2 $$\begin{aligned} T_i = I_w \cdot (w_{1} \cdot T_{IP_{left}} + w_{2} \cdot T_{IP_{right}}) \end{aligned}$$

with $T_i$ representing the temperature of the current voxel *i* and $T_{IP_{left}}$, $T_{IP_{right}}$ representing the temperature of the adjacent interpolation partners. To reduce the background noise caused by the air outside of the phantoms, the data sets were cropped to a 60x60mm region of interest (ROI). The morphological opening to reduce the remaining background noise was replaced in this version by a connected component analysis. An example of the method from coronal, axial and sagittal view can be seen in Fig. 1.

### Outlier detection

For the Delaunay trinagulation, the MVEE and the splines method an outlier detection was developed to improve the robustness towards single outlier slices during image acquisition. For this approach we compare the latest acquired image at time point $t_i$ to the previous time point $t_{i-1}$ for that specific orientation with the assumption that our coagulation necrosis is always increasing in size and never shrinking. First, a connected component analysis is performed to remove background noise and small irregularities. Second, the growth of the coagulation necrosis $\Delta A$ in percentage is computed and compared between both time points using:

3 $$\begin{aligned} {{ \Delta A = \frac{|A_{t_i} - A_{t_{i-1}}| \cdot 100\%}{A_{t_{i-1}}} }}\end{aligned}$$

with *A* representing the area of the coagulation necrosis. If $\Delta A > 80\%$ an abnormal behaviour was detected. This specific value was defined empirically by observation of all phantoms. If a slice is considered an outlier this slice is not taken into account for the current reconstruction.

### Delaunay triangulation

By considering the necrotic voxels as a point cloud, a triangulation can be performed to get an outer hull. This hull features a global and smooth connection of edges resulting in the contour of the estimated coagulation necrosis. In addition, staircase artifacts due to consideration of only single slices are prevented and the polygon mesh is more suitable for post-processing and visualization.

A standardized method for generating a closed surface mesh from a set of points is the Delauny triangulation. It divides an unstructured point set into an uneven triangular grid. To compute the triangulation, we utilize the VTK implementation based on the works of Watson^23^ and Bowyer^22^. Their algorithm is based on an incremental approach, where one point is added each time to an already valid Delaunay triangulation. Therefore, an initial triangle is placed inside the triangulation, which is big enough to enclose all points of the initial point cloud. These points are now added one after another to the triangulation. Every time this is done, all invalid triangles are identified. For these triangles, the polygonal hole is identified and the triangles are removed from the data structure. After removal, the polygonal hole is re-triangulated and the next point from the input point cloud is inserted in the triangulation. Finally, every triangle is determined if it contains a vertex, which is part of the initial triangle. If a vertex is found, the corresponding triangle is removed from triangulation as well. The approach from Watson and Bowyer also introduce a few acceleration steps which cause the algorithm to be faster than the original method. After computation of the modified Delaunay triangulation a convex hull is generated as an output. For creating a volumetric necrosis zone, the voxels within the surface mesh are also marked as necrotic. An example of the Delaunay reconstruction can be seen in Fig. 1.

### Minimum volume enclosing ellipsoid

The construction of an ellipse around the applicator’s main axis models the idealized concentric heat distribution. The applicator can be interpreted as many heat point sources in a row. The maximum of heat is generated in the electrode of the applicator and decreases along the applicator’s main axis. In homogeneous media, the heat of a plane point source decreases exponentially with quadratic distance. This results in the typical ellipsoid shape of the coagulation necrosis. With this a priori knowledge, a proper geometric model is determined. In our approach, ellipses are formed slice-wise perpendicular to the applicator’s main axis following the extent and alignment of the necrotic voxels. By exploiting this behaviour, irregularities of the heat distribution or incomplete data can be compensated by the convex hull.

The implementation is based on the work by Nima Moshtagh’s algorithm for the computation of the MVEE^31^. This algorithm calculates the parameters of an ellipsoid with the smallest volume by containing a set of *n* dimensional data points $P_i$. The algorithm is applied slice-wise on each 2D voxel slice perpendicular to the applicator’s main axis. The parameter consisting of the center c, the two radii $r_1$ and $r_2$ and the rotation matrix of the ellipse are calculated by solving the following optimization problem:

4 $$\begin{aligned} log(det(A)) \xrightarrow {} min \end{aligned}$$

such that

5 $$\begin{aligned} (P_i - c)^\intercal \cdot A \cdot (P_i -c) \le 1 \end{aligned}$$

with *A* containing all information regarding the shape of the ellipses. This information can be decomposed by a singular value decomposition:

6 $$\begin{aligned} {[}U\, Q\, V] = svd(A) \end{aligned}$$

with *U* and *V* defining a first and second rotation matrix of the ellipse, respectively. The scaling matrix *Q* is containing the singular values $\sigma _1$ and $\sigma _2$, which are representing the semi-major and semi-minor axis of the ellipse. The radii $r_i$ can now be calculated by:

7 $$\begin{aligned} r_i = \frac{1}{\sqrt{Q_{ii}}} \end{aligned}$$

**Figure 2:**
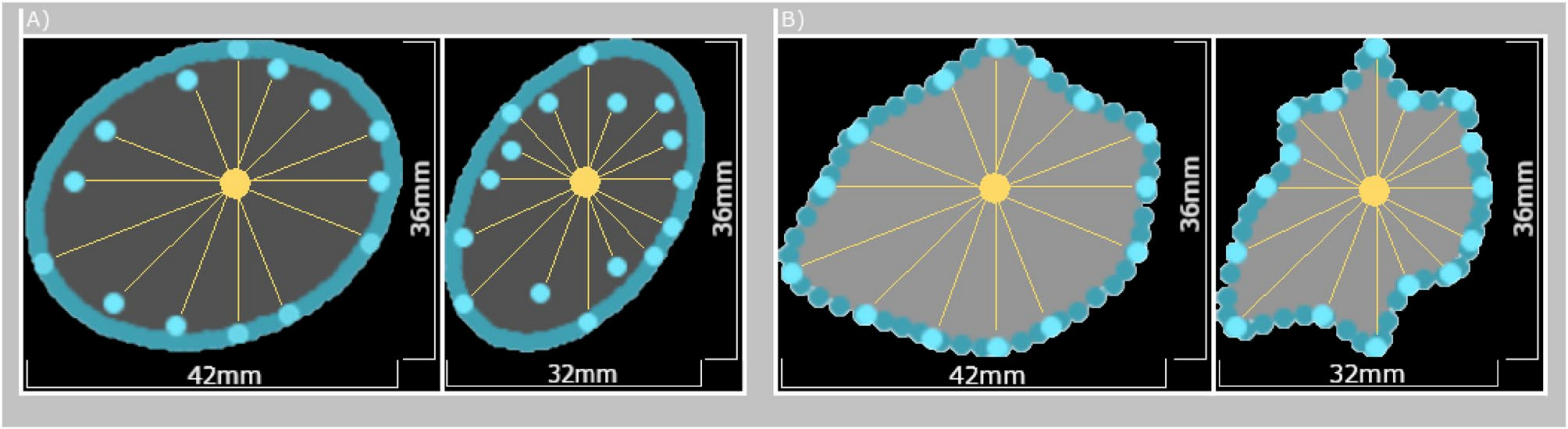
MVEE (**A**) and Spline (**B**) reconstruction for homogeneous phantom number 1. Exemplary shown are slice 44 and 58 along the applicator’s main axis (yellow dot). Visible are the rotated MR images (yellow lines), the data points used as an input (light turquoise) and the corresponding computed outline of the reconstruction (dark turquoise). Note that the number of input points may vary due to outlier detection.

Each voxel $x_i$ inside of the generated ellipses is assumed to be coagulated and will be flagged as necrotic. In this way, a three-dimensional coagulation necrosis is created. An example of two slices along the applicator’s main axis can be seen in Fig. 2.

### Splines

Considering the necrotic contour voxels of the live data slice-wise perpendicular to the applicator’s main axis, the link between two neighbored voxels is done by a polynomial of third degree for creating a smooth, naturally curved shape. This method forms a concave and closed hull. The effect of drawing a tight hull around the data only results in a local impact of outliers on the overall reconstruction. The interpolation of incorrect live data is determined by the two neighbored slices. Hence, a proper sampling rate of the volume leads to a good reconstruction despite erroneous or missing data.

The 3D volume consisting of the oriented necrosis maps is sliced perpendicular to the applicator’s main axis. The order of connecting the voxels *V* is determined by the size of angles regarding their cylinder coordinates $V_c$:

8 $$\begin{aligned} V(x,y,z)&= V_c(r, \theta , z)\nonumber \\ r&= \sqrt{\left( x-x_c \right) ^2 + \left( y-y_c\right) ^2}\nonumber \\ \theta&= atan2\left( \frac{x-x_c}{y-y_c}\right) \end{aligned}$$

with (*x*, *y*) representing the Cartesian coordinates of a voxel in a slice *z*, *r* representing the radius, $\theta$ representing the angle of the cylindric coordinates and $(x_c, y_c)$ representing the Cartesian coordinates of the applicator’s main axis in each slice *z*. The voxels are listed in ascending order of their angles in relation to $0^\circ$ MRI slice, so that for two voxels *i* and *j*, the following neighborhood condition applies:

9 $$\begin{aligned} 0 > i< j \le n \text { if }\theta _i < \theta _j \end{aligned}$$

Afterwards, the set of voxel data is interpolated by a continuous closed Cubic Bezier Curve using de Boor’s Algorithm^28^. De Boor’s algorithm introduces a fast and stable way to evaluate a point on a B-spline curve by not directly computing the B-spline functions but evaluating the spline curve through an equivalent recursion formula. For determining the voxels inside the closed hull to flag them as necrotic, the winding number algorithm after Sunday’s implementation^32^ is used. This is more robust for points close to complex polygon boundaries and is as fast as comparable methods. An example of the splines method for two slices is shown in Fig. 2.

## Experimental setup

**Table 2:** Summary of the ANOVAs’ results. Df = Degrees of Freedom in the numerator, F = F-value, p = probability of the data given the null hypothesis, Sig. = p-values less than the traditional $\alpha$ <0.05, $\eta ^2$ = Generalized Eta-Squared measure of effect size.

Variable	*df*	F	*p*	Sig.	$\eta ^2$
**Accuracy**					
Algorithm with Global Threshold	3	3.86	0.017	*	0.056
Algorithm with Median Threshold	3	4.16	0.013	*	0.107
Algorithm with Local Threshold	2	51.82	<0.001	*	0.542
Algorithm with Ground Truth Threshold	2	116.66	<0.001	*	0.687
**Adaptability**					
Algorithm with Median Threshold	3	39.99	<0.001	*	0.81

Evaluation of the explained reconstruction methods was conducted on the same 13 bio protein data sets as introduced in our previous work^8^ to ensure comparability of the results. The phantoms are created according to Bu Lin et al.^33^. Seven out of these 13 phantoms were homogeneous while six had PVC tubes (diameter = 5 mm, wall thickness = 1 mm) inserted to simulate a possible heat sink effect. To verify the temperature accuracy, two of the homogeneous phantoms had temperature sensor inserted. In addition to the paper of Bu Lin et al.^33^, additional contrast agent ($0.5\,\upmu \mathrm{mol}/\mathrm{L}$ Dotarem) was added for better visibility of the coagulation necrosis in the post treatment 3D turbo spin echo data sets used for manual ground truth segmentation of the coagulation necrosis by a medical expert. All images were acquired on a 1.5T MR scanner (Siemens Avanto, Siemens Healthineers, Germany) and MWA ablation (MedWaves Avecure, Medwaves, San Diego, CA, USA, 14G) was performed with a maximum antenna power of 36W, a maximum temperature of $90\,^\circ$C and a duration of 15  min. A detailed explanation of the whole image acquisition protocol is described in our previous work^8^. We evaluated all methods with respect to:

1. Accuracy: Improvement of the Dice Score Coefficient (DSC) and the Squared Error of the Mean (SEM).
2. Robustness: Compensation of artifacts and MR inhomogeneities.
3. Adaptability: Necessity of a priori knowledge about vessels and real-time capability.

If not mentioned explicitly, all results were acquired without taking the a priori knowledge about vessels into account. All data sets provide a ground truth manually extracted by a medical expert after the intervention. The DSC was computed according to our previous work^8^ using an SEM at a confidence level of 95% (*p* = 0.05) over all 13 phantoms. In the following sections outliers will refer to single slices in the interventional protocol, which show a very low signal to noise ratio. This causes the predefined threshold to highly overestimate the coagulation necrosis even when optimized. With respect to the originally published temperature interpolation these corrupted slices caused the method to fail. Therefore, these perfusion phantoms 1 and 4 are examined carefully during the evaluation. Examples can be seen in Supplementary Fig. 2.

**Figure 3:**
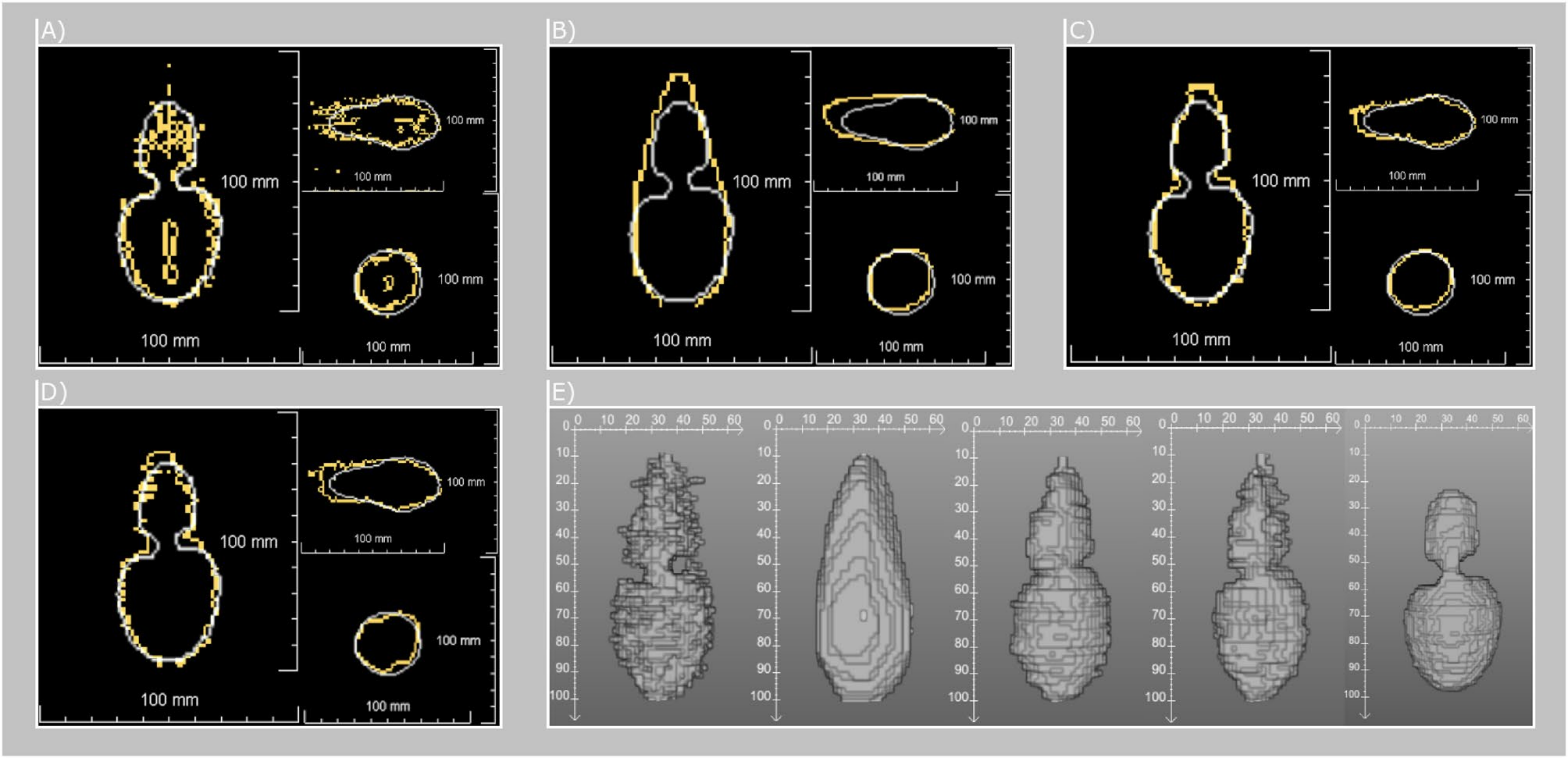
Reconstructions for perfusion phantom 2. Visible are the ground truth contours (white) in addition to the output contour (yellow) in axial, sagittal an coronal. (**A**) Temperature Interpolation. (**B**) Delaunay triangulation. (**C**) MVEE. (**D**) Splines. (**E**) 3D representation without smoothing for all methods including the ground truth at the right.

During testing of the accuracy, we had a closer look at the introduced bias for estimating the coagulation necrosis. Due to the use of bio protein phantoms we decided to apply the critical temperature model instead of the CEM43 or Arhenius model^34,35^ . This thermal threshold was identified for each phantom individually within a range of 50°–60°. Because the evaluation is focused on the reconstruction algorithms and not on the temperature accuracy of the acquired images the used coagulation thresholds were identified by applying the thresholds in a range from 0° to 100° for each orientation. The results for each value were compared to the corresponding image plane in the ground truth and the optimal threshold for each orientation was identified. Due to the pH-value inhomogeneities in the phantoms this approach shows different optimal thresholds for different orientations reflecting the tissue inhomogeneities in a human. Unfortunately, the phantoms did not show a proper sub-lethal transition zone in the post-treatment images. Therefore, we only applied one threshold to define if a voxel is coagulated or not, neglecting sub-lethal damage to the tissue. Nonetheless, the threshold for this estimation highly depends on the pH-value of the phantoms, which can vary much within each phantom according to Bu Lin et al.^33^. To address this bias, we not only evaluate our new methods with the original global threshold used in our previous work^8^, but also introduce two other thresholds. In addition, we want to investigate how well our methods will perform considering perfect input. Therefore, we resliced the given ground truth for each data set and extracted the eight predefined orientations usually acquired during live imaging. Afterwards, these eight slices are used as an input for reconstruction to simulate a perfect input. The resulting set of tested inputs consists of the following three thresholds for necrosis estimation and the resliced ground truth:

1. Global: Original threshold used in our previous work.
2. Median: The optimal threshold for each orientation was empirically determined. Afterwards, the median threshold value from each of the eight orientations was used for reconstruction to reliably remove outlier thresholds (global compensation of pH value inhomogeneities , which may result in unrealistic thresholds of e.g, $25^\circ$ or $84^\circ$ ).
3. Local: The optimal threshold for each orientation is used for reconstruction (local compensation of pH value inhomogeneities).
4. Ground truth: Resliced ground truth offering perfect input.

To test the robustness of our methods, we focus on the evaluation of phantoms with perfusion to simulate a heat sink effect. Here, the perfusion phantoms number 1 and 4 show strong artifacts and MR inhomogeneities causing our initial method to fail. We compare all our methods with and without these critical data sets to determine the influence of heavily corrupted data on our methods. Regarding the adaptability, we performed each reconstruction two times. First, we take the a priori knowledge into account by providing the segmented vessel structures as input to each algorithm. After reconstruction, these vessels are subtracted from the result to enforce a proper deformation of the reconstructed volume. During the second reconstruction, no a priori knowledge is provided as additional input. For evaluation, we computed the volume of false positive classified voxel values by subtraction of both corresponding reconstructions. Thus, we are able to identify which algorithm performs best in the presence of vessel structures. In addition, each reconstruction was performed 100 times to measure the mean computational time and the standard deviation. For the accuracy and adaptability experiments, additional one-way ANOVAs were conducted with respect to the different used algorithms. Afterwards, post-hoc tests were performed using pairwise *t* tests with Bonferroni correction. An exemplary overview about the performance of the algorithms tested can be seen in Fig. 3.

## Results

We evaluated our methods regarding accuracy, robustness, and adaptability. In the following subsections, we present our results with respect to these three parameters. A summary of the ANOVAs’ results can be observed in Table 2. Statistically significant post-hoc test results can be seen in the corresponding Figures for accuracy and adaptability tests. All boxplots show the interquartile range (box), the 25th and 75th percentile (borders of each box), the median (horizontal line in the box), the minimum and maximum values (whiskers) and the potential outliers (dots).

**Figure 4:**
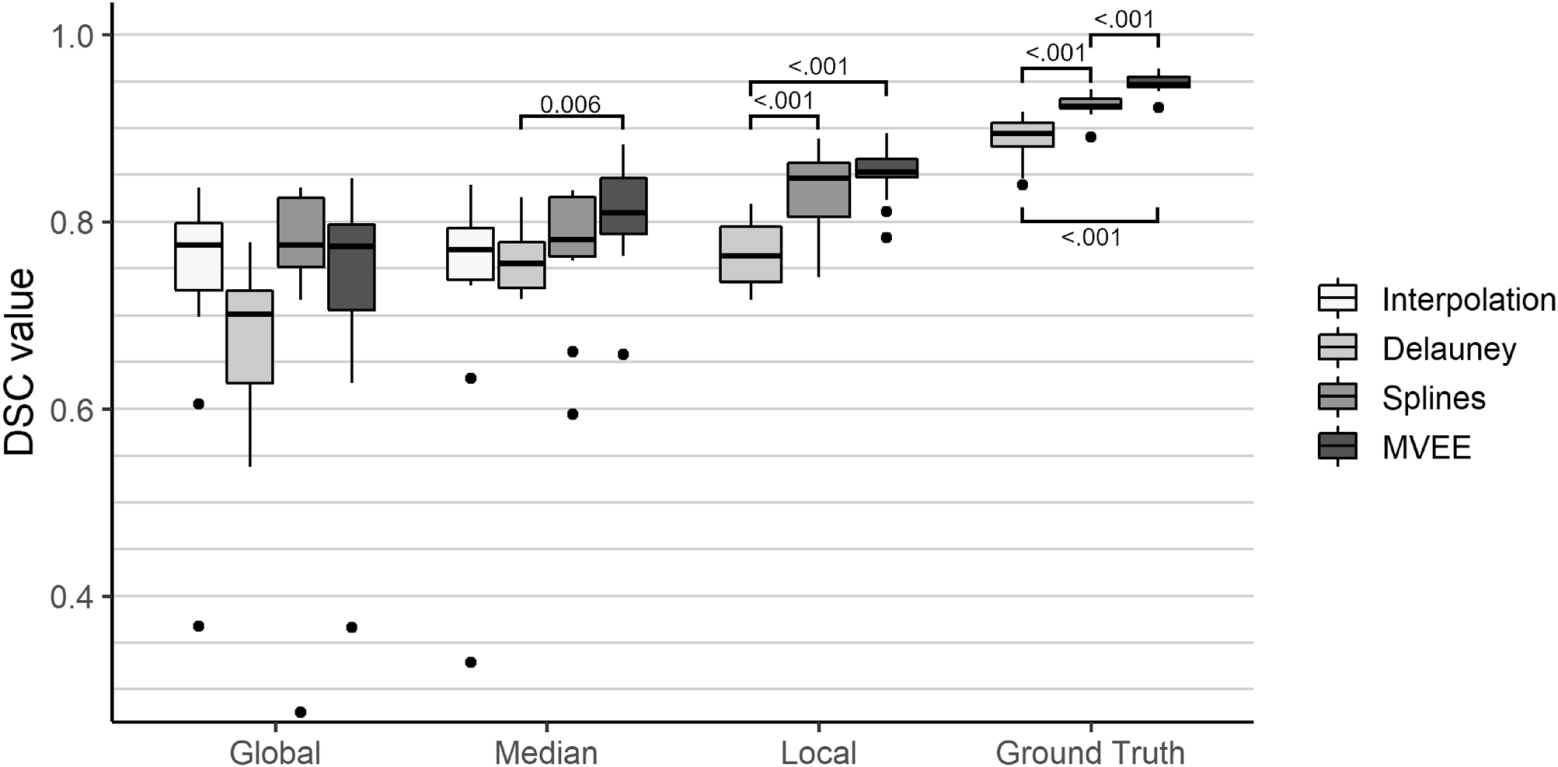
Results of the accuracy tests for all 13 phantoms. DSC measurements are separated for each method and each tested threshold with: Global = Threshold used in our old approach. Median = Median threshold from all eight orientations. Local = Individual threshold for each orientation. Ground Truth = Resliced input data from the ground truth. Horizontal lines indicate statistically significant post-hoc pairwise *t* test results.

### Accuracy

An overview about our accuracy test results can be observed in Fig. 4. The global threshold is identical with the one used in our previous work^8^. The splines ($0.75\pm 0.08$) and MVEE ($0.74\pm 0.07$) method yield comparable DSC results to the original temperature interpolation ($0.73\pm 0.07$) method, whereas the Delaunay triangulation performs more poorly ($0.69\pm 0.04$). The median thresholds for each data set shows slightly different results. The effect of the new thresholds mainly affects the corrupted data sets of the perfusion phantom 1 and 4. Here, the compensation of the bias introduced by the pH value variations causes the splines ($0.77\pm 0.04$), MVEE ($0.80\pm 0.03$) and even the Delaunay triangulation ($0.75\pm 0.02$) to outperform our previous method ($0.73\pm 0.07$). The local threshold, which is individual for each of the eight orientations, could not be applied to our previous method because the necrosis estimation was performed on the reconstructed volume instead of every input slide. For the new approaches, the optimized local threshold shows that the Delaunay triangulation ($0.78\pm 0.02$) performs more poorly than the other two. The splines method ($0.81\pm 0.02$) performes somewhat worse than the MVEE ($0.84\pm 0.02$) regarding the mean DSC, but still better than Delaunay and the temperature interpolation method.

Overall, the results regarding accuracy show a meaningful trend in favor of the MVEE method followed by the splines approach. The Delaunay triangulation performs worst in most tested scenarios. This statement is supported by evaluation of the ground truth reconstruction which simulates a perfect input. Here, the Delaunay triangulation achieves a maximum of $0.88\pm 0.02$, the splines of $0.92\pm 0.01$ and the MVEE of $0.95\pm 0.01$. For the purpose of further testing, the median threshold will be used, as it best reflects the real clinical conditions.

### Robustness

**Figure 5:**
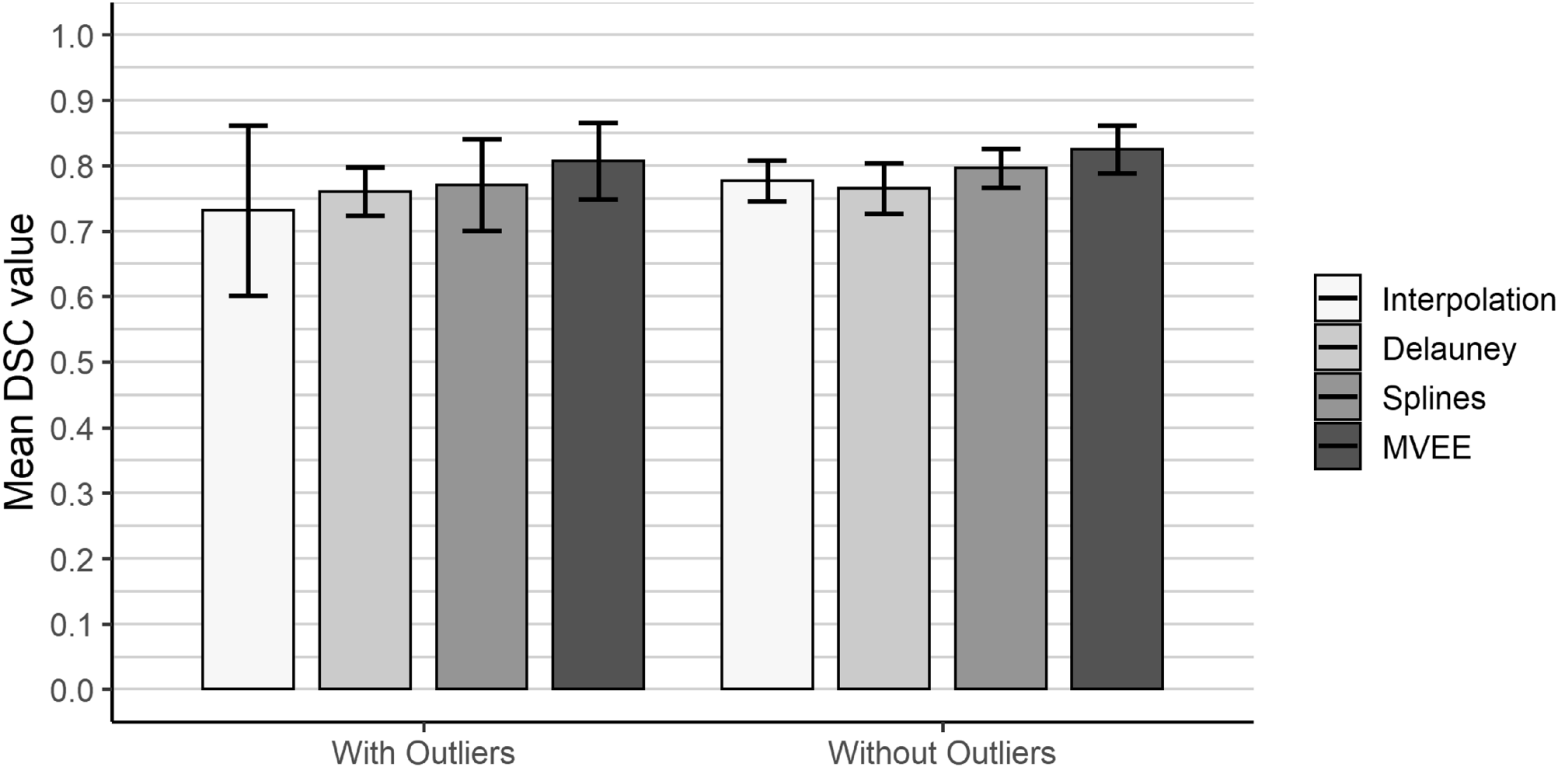
Results of the robustness tests. The median threshold was used for necrosis estimation and the error bars correspond to the standard deviation. With outliers = All data sets were taken into account including the highly corrupted data sets. Without outliers = Perfusion phantom 1 and 4 were left out of the evaluation. No statistical significance was found.

A graphical overview about the results regarding the robustness test can be seen in Fig. 5. After removing the corrupted perfusion phantoms number 1 and 4, the mean DSC of the interpolation method is improved by 0.05, while the standard deviation decreases by 73.61% from $\sim$0.07 to $\sim$0.02. The Delaunay triangulation improves by just 0.01 with a nearly identical standard deviation. The spline method shows a 0.04 higher DSC after outlier removal, whereas the MVEE method shows an improvement of 0.02. Regarding the standard deviation, the splines method improves by 53.87% from $\sim$0.04 to $\sim$0.02 and the MVEE method by 32.07% from $\sim$0.03 to $\sim$0.02. We performed two sample t-tests between the groups “With Outliers” and “Without Outliers” for the algorithms temperature interpolation ($p=0.27$), Delauney ($p=0.75$), Splines ($p=0.27$) and MVEE ($p=0.40$). No significant differences could be observed.

Overall, we can observe that the new methods show less variation in the standard deviation, including the corrupted data sets than the original temperature interpolation method. Nonetheless, no significance could be observed. Between the new approaches, no trend can be observed.

### Adaptability

**Figure 6:**
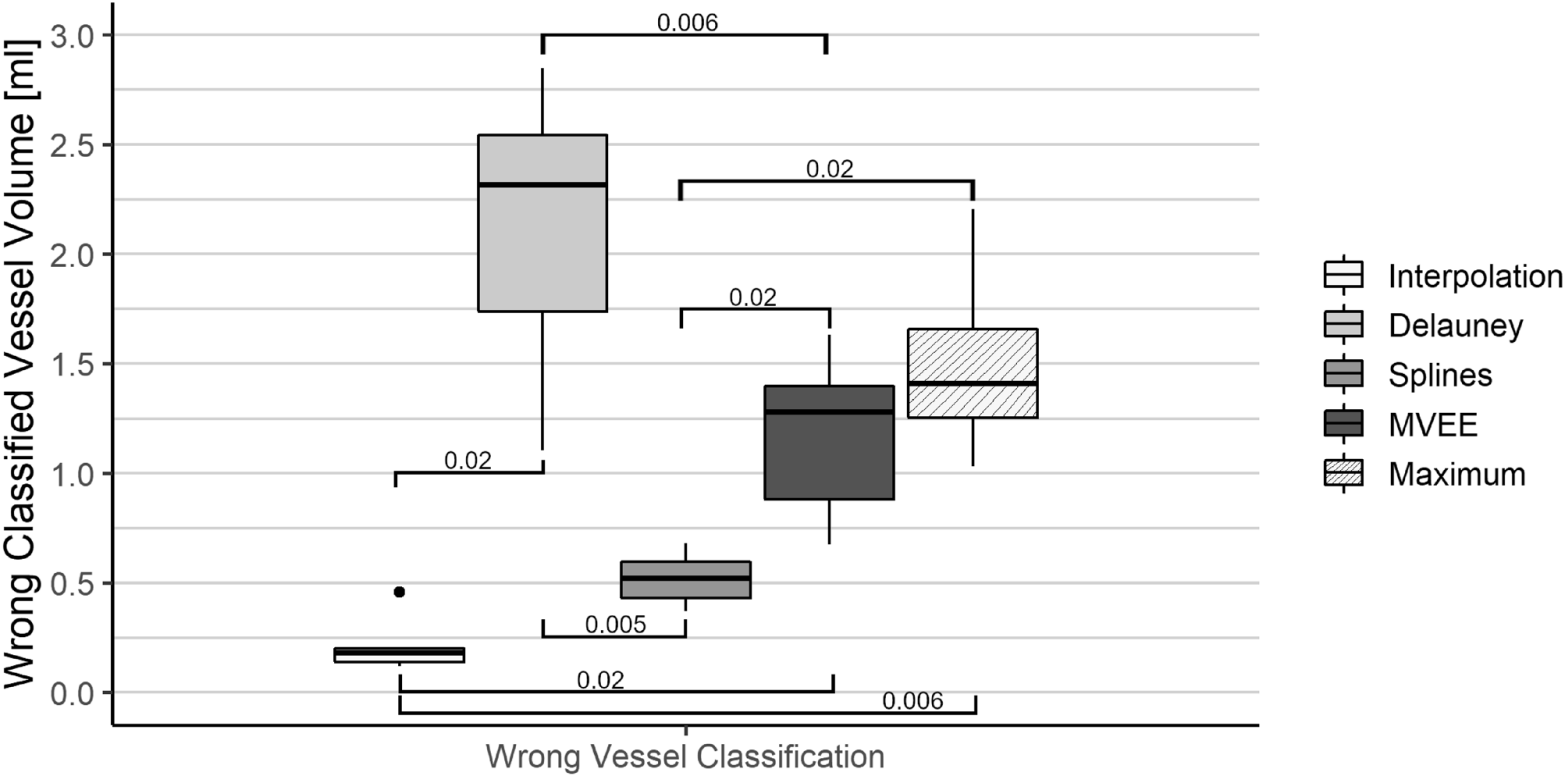
Results of the adaptability tests. The volume of the wrongly classified voxels can be seen for each of the four algorithms. In addition, the boxplot “Maximum” indicates the true vessel volume affected by the coagulation necrosis over all six perfusion phantoms. Horizontal lines indicate statistically significant post-hoc pairwise t-test results.

An overview of the adaptability test can be observed in Fig. 6. The original interpolation method shows a mean volume of $0.21\pm 0.10$ ml incorrectly classified voxels, closely followed by the splines and MVEE method with $0.52\pm 0.10$ ml and $1.18\pm 0.30$ ml, respectively. The Delaunay triangulation offers the worst result regarding the adaptability with a mean volume of $2.12\pm 0.53$ ml false positive classification regarding the vessels. The maximum vessel volume affected by the coagulation necrosis was computed at $1.5\pm 0.42$ ml.

Overall, it can be said that the original interpolation method shows the least false positive classification of vessel voxels whereas the Delaunay triangulation performs the worst regarding the vessels even exceeding the maximum vessel intersection due to overestimation of the coagulation necrosis. While comparing the splines and the MVEE method, we noticed that the splines not only showed a lower mean for false positive classification, but also provided a much lower standard deviation over all six perfusion phantoms. Regarding the robustness there is a trend towards the temperature interpolation and the splines.

With respect to the reconstruction times, the temperature interpolation (C++) method shows $8.02 \,{\rm ms} \pm 5.91\, {\rm ms}$, the Delauney trinagulation (Python) $1.57\,{\rm s}\pm 0.3\,{\rm s}$, the MVEE method (Python) $4.5\,{\rm s}\pm 0.6\,{\rm s}$ and the Spline method (Python) $6.2\,{\rm s}\pm 0.55\, {\rm ms}$.

## Discussion and conclusion

Regarding accuracy, our results show a strong trend in favor of the MVEE method. Apparently, MR inhomogeneities and other artifacts cause our data to frequently underestimate the coagulation necrosis when computed through a simple threshold. In these cases, the MVEE method is able to compensate for this loss of information and provide a proper reconstruction. The big disadvantage of the MVEE approach, on the other hand, can be observed during experiments with the perfusion phantoms. The compensation of the underestimation of the coagulation necrosis also causes the vessels to vanish to an extent, which is not considered negligible. The splines method was shown to be more capable of dealing with vessel structures, but on the other hand shows a lower DSC similarity overall. The Delaunay triangulation shows an even worse performance than our original method, which is caused by the approach itself. Fitting a triangulation mesh to a set of points completely neglects any deformation caused by vessels and others. Even though this approach is well suited for homogeneous heat distributions without any anomalies, it is far from a real-life scenario. Therefore, the Delaunay triangulation will not be investigated further.

The differences regarding the similarity are mainly caused by a continuous underestimation of the coagulation necrosis. This underestimation is not only caused by the approach used to recompute the heat maps, the noise, and other artifacts in the images, but also by the partially strong pH value variations within the phantom. Here, it would be suitable to conduct more studies with ex vivo organs to see if these variations have an impact on the overall similarity of reconstruction. In a later step, our image acquisition protocol should also be tested in a real clinical setup, e.g., with the use of in vivo animal experiments. In addition, the adaptability of our approach may be increased by a dynamic framework which does not need predefined reference images for heat map reconstruction. To achieve this reference-free thermometry, approaches as introduced by Salomir et al.^36^ can be combined with our framework. A reference-free thermometry would allow for a life adaptation and change of the region of interest, e.g., when a vessel or organ boundary was automatically detected during the intervention (e.g., through deep-learning approaches). To overcome the temporal heat variation in single voxels caused by local MR inhomogeneities or artifacts, it might be suitable to combine our approach with real-time simulations of the heat transfer. A first approach would be the use of the Pennes’ Bioheat Equation^37^ while optimizing the simulation parameters based on our live data for different orientations. This approach could result in an even higher accuracy while providing a visually more appealing result, which can be easier to use by a clinical end user. Furthermore, this approach may also be extended by using a Kalman Filtering to achieve a self-adaptive hybrid magnetic resonance thermometry as introduced by Zhang et al.^38^. Their method offers the possibility to follow temperature changes in presence of motion and adapt the temporal and spatial resolution of the thermometry. Using Kalman filters the acquired data can be corrected, and corrupted data points within the images can be removed. Therefore, this approach may be beneficial to better spot heat sinks (e.g. caused by vessels) and get a better understanding of their effects during ablation.

Using the resliced ground truth, we showed that our methods works well with appropriate initial input, which is mainly dependent from the used 2D GRE sequence. Because our setup is able to take any 2D phase or temperature input with any number of orientation, we can directly utilize new image sequences as soon as they are published. This allows our setup to be applicable to a wide range clinical setups as well as providing an improved reconstruction along with the improvement of the MR sequences.

**Conclusion** In this work, we presented three new approaches for the reconstruction of a volumetric coagulation necrosis for the monitoring of MWA procedures. All methods are able to utilize any 2D MR sequence, as long as the sequence provides phase images for heat map reconstruction. We were able to show that our spline and MVEE approach have the potential for highly accurate reconstruction of the volume while outperforming our originally proposed method in a more realistic setup regarding accuracy and robustness. To overcome local inhomogeneities caused by noise or MR dependent artifacts, the use of adaptive simulations should be considered in the future to compute a more homogeneous volumetric map. Future work should also conduct studies in ex vivo and in vivo animal experiments to verify transferability from the phantoms to a more realistic environment.

## Supplementary Information

Supplementary Information.

## Data Availability

The data sets processed and analysed during the current study are available in the Open Science Repository for Research Data and Publications of OVGU (Creative Common License 4.0), http://open-science.ub.ovgu.de/xmlui/handle/684882692/89?locale-attribute=en. In addition, the datasets generated during and/or analysed during the current study are available from the corresponding author on reasonable request. The source code used for generating the results presented in this study are publicly available via https://github.com/jalpers/ScientificReports2022_ComparisonStudy.
