# Supplementary material for: Comparison study of reconstruction algorithms for volumetric necrosis maps from 2D multi-slice GRE thermometry images
Source: Sci Rep. 2022 Jul 7;12:11509. doi: 10.1038/s41598-022-15712-7 (PMC9263155; doi:10.1038/s41598-022-15712-7)
Supplement: Supplementary file 1 — Supplementary Information. [file 41598_2022_15712_MOESM1_ESM.pdf]

Comparison Study for Reconstruction Algorithms of Volumetric Necrosis Maps from 2D Multi Slice GRE Thermometry Images – Supplementary Information

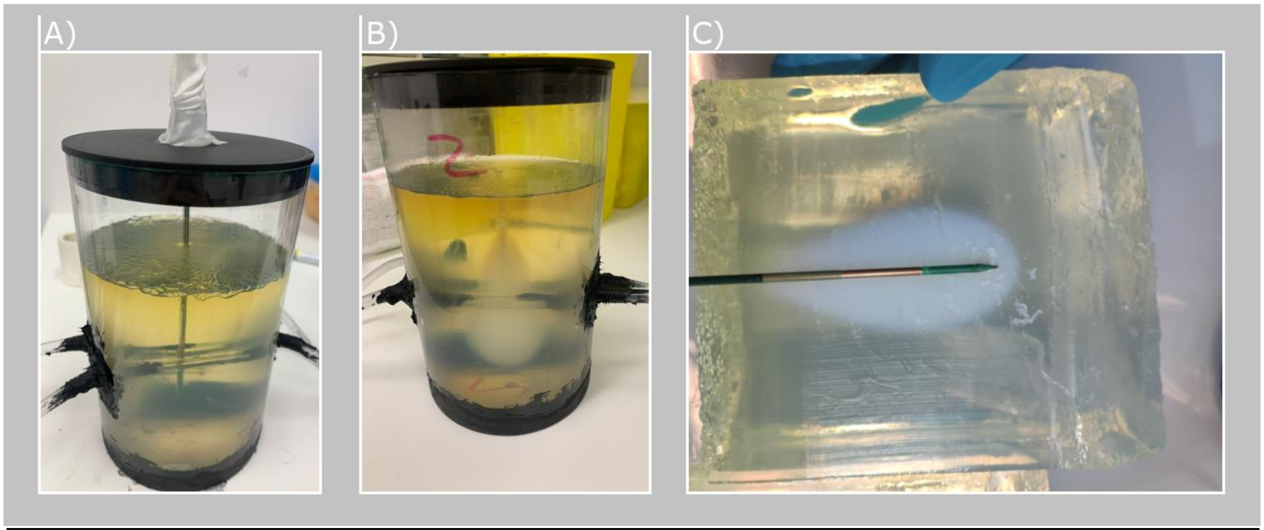

Supplementary Figure S1. A) Photography of the used polyacrylamide phantom with two inserted PVC tubes before ablation. B) The same phantom after ablation. The PVC tubes, perfused with water, cause a heat sink effect resulting in an inhomogeneous coagulation necrosis (opaque drop inside the phantom. C) Profile of a homogeneous phantom after ablation with MW applicator as reference.

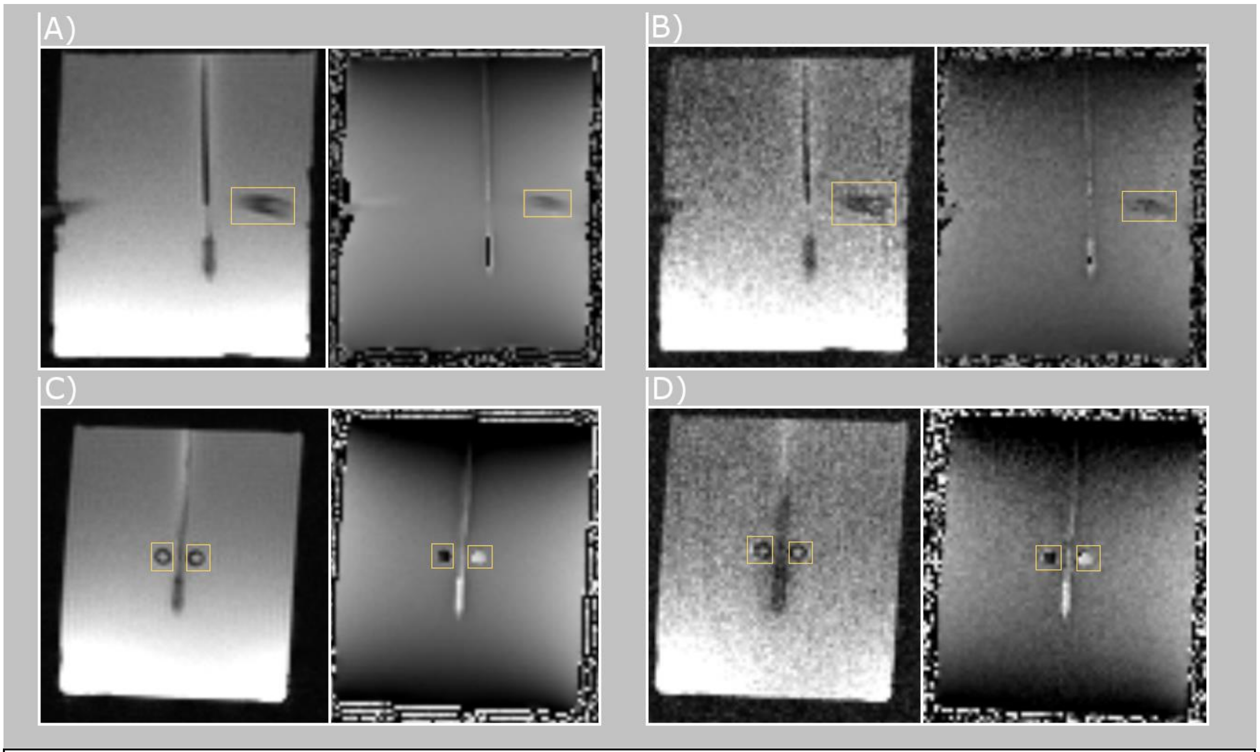

Supplementary Figure S2. All four panels show the magnitude (left) and corresponding phase (right). A) Artifact affected perfusion phantom 1 at time point 1. B) The same phantom at time point 2. C) Artifact affected perfusion phantom 4 at time point 1. D) The same phantom at time point 2. It is noticeable that the SNR of the data changing rapidly between two consecutive time points. The yellow rectangle show the inserted PVC tubes.
